# Team leader communication in ad hoc teams and its impact on team outcomes: a systematic review

**DOI:** 10.1186/s12913-025-12887-3

**Published:** 2025-06-02

**Authors:** Oddveig Reiersdal Aaberg, Dag Tomas Sagen Johannesen, Ellen Benestad Moi, Gro Frivold, Fredrik Jensen, Kristine Haddeland

**Affiliations:** 1 University of Agder, Campus Kristiansand, Universitetsveien 25, Kristiansand, 4630 Norway; 2 University of Agder, Campus Grimstad, Jon Lilletuns vei 9, Grimstad, 4879 Norway; 3 Sorlandet Hospital Trust, Egsveien 100, Kristiansand, 4615 Norway

**Keywords:** Action team, Emergency team, Hospital, Team leader communication, Team effectiveness, Team outcome, Team performance

## Abstract

**Background:**

Ad hoc teams often work on acute medical problems in intense and unpredictable situations. Team leadership is important for effective teamwork, and communication in ad hoc teams is especially vulnerable to errors and may impact patient outcomes. Previous review studies have mainly focused on team leadership styles. However, little is known about team leader communication in ad hoc teams and whether it impacts team outcomes. Thus, the aim of this review was to investigate whether team leader communication in ad hoc teams impacts team outcomes.

**Methods:**

A systematic review was conducted with database searches on CINAHL, Medline and Embase in May 2023, with an update in February 2024. Eight studies identified from the search were included in the review, and the results are presented as a narrative synthesis.

**Results:**

The eight studies that met our inclusion criteria had an observational quantitative design and all of them were conducted in the context of emergency departments or intensive care units (five from a simulation context and three from a real life context). Team leader communication was measured using validated instruments or behavioural indicators, such as call-outs and closed loops. The results revealed that team leader communication had a significant impact on team performance, measured as technical work performance and non-technical work performance, in all the included studies. The team leaders’ use of communication strategies, such as situational awareness, callouts and closed loop communication, positively impacted team performance.

**Conclusions:**

The results of this systematic review indicate that team leaders in ad hoc teams have a significant impact on overall team performance. These results underscore the importance of competence in team leadership and highlight the importance of incorporating team leader training into team training programmes. Further studies using more homogenous measuring methods for both team leader communication and outcome variables are needed to confirm the results of this review. The findings in this review may contribute to an enhanced emphasis on the training of team leaders in intense, unpredictable situations, such as in ad hoc teams.

**Trial registration:**

PROSPERO: CRD42023430082.

## Background

Failures in teamwork and communication have been identified as the most common causes of adverse events in hospitals [1]. Communication breakdowns that cause adverse events can have fatal consequences for patients [2]. In ad hoc teams, in which there is little time for planning, effective teamwork is crucial [3, 4]. Hence, team leadership is important for team outcomes, and communication with team members is the main task of team leaders [5]. 

Ad hoc teams are composed of members with specialised skills who are required to improvise and coordinate their actions in intense, unpredictable situations [6]. In such teams, team members change across shifts and rotations and typically work in the emergency department (ED), intensive care unit (ICU) and operating room (OR) [7]. Team members deal with patients in unexpected crisis situations, such as trauma, acute deteriorations and complications and cardiac arrest [3]. The team composition is typically interprofessional, including nurses, physicians and other professionals, with a designated team leader [3]. Team members work independently, which means that they cooperate and work interactively to complete tasks [8].

Team leader communication plays a key role in teamwork in ad hoc teams. Open and effective communication depends on effective leadership and information sharing [9] and requires using a calm and clear voice and stating commands clearly [10]. A team leader is supposed to monitor and modify plans, communicate changes and provide feedback on their team’s performance [11, 12]. Effective leadership may be achieved when the team leader uses communication strategies, such as closed loop communication (CLC), to achieve and maintain a shared mental model (SMM) to improve outcomes [13–15]. Interpersonal communication, feedback and shared decision-making are typical leadership behaviours in ad hoc teams [16]. Various instruments have been developed to measure team leadership. Despite the expansion of teamwork and leadership training, reports of poor leadership in healthcare action teams persist in both clinical and simulated emergencies [17]. 

Team performance and other types of team outcomes are common variables of interest in the research of team effectiveness, as teamwork is defined as the production of useful outcomes in an organisation [8]. Team outcomes can be described as team performance and have been measured in various ways in previous studies [8]. Hiller et al. [18] measured team performance as team effectiveness and included planning, problem solving, support and consideration in the definition of team effectiveness. Others have measured team effectiveness as quality, overall achievement, productivity, knowledge and interpersonal skills [8]. Non-technical skills (NTS) are defined as cognitive, social and interpersonal skills [19]. So-called technical skills, also described as clinical work, are used in combination with NTS to manage tasks efficiently and safely.

A systems structure involving input, process and outcome can be used to understand the influence of team leader communication in the team process and thereby how it influences the team performance [8]. Team outcomes can be measured as team performance based on technical work performance or non-technical work performance [20, 21]. Inputs are conditions that exist prior to teamwork processes, while processes are the team leader’s and members’ actions and interactions [22]. 

Research outside the field of healthcare has demonstrated that team leadership can positively predict team performance [23, 24]. Previous research on hospital teams has indicated that leadership behaviour may affect team members’ work performance [25–28] and team outcomes [29–32].

Most reviews of team leaders’ impact on team performance in healthcare have focused mainly on healthcare teams in general [27, 28, 33, 34]. A meta-analysis revealed that task-focused behaviours were moderately linked to perceived team effectiveness and productivity, while the person-focused behaviours of team leaders were associated with perceived team effectiveness, productivity and learning [28]. 

A review of leadership in ad hoc critical care teams identified three types of leadership but found little evidence of leadership styles contributing to improved team outcomes [17]. In their review of leadership strategies in critical care teams, Künzle and colleagues [35] found that effective leadership plays a crucial role in promoting team performance and patient safety. Previous research on ad hoc teams, such as cardio pulmonal resuscitation (CPR) teams and trauma teams, indicates that effective team leadership is associated with improved team outcomes [36]. However, little is known about whether a team leader’s communication in ad hoc teams influences team performance. The current review supplements previous reviews by focusing more narrowly on team leaders’ communication and its impact on team performance. Therefore, the aim of this review was to investigate whether team leader communication in ad hoc teams impacts team performance. The insights gained from this review can be used to train team leaders, thereby improving team performance in ad hoc teams.

## Methods

This systematic review was a review of observational quantitative studies conducted in accordance with the Preferred Reporting Items for Systematic Reviews and Meta-Analyses (PRISMA) checklist [37]. The study protocol was registered in PROSPERO [38]. 

### Search strategies

Appropriate keywords related to leader, leadership, team, communication, performance, and acute settings in various combinations were identified to plan the literature search (see Table 1). The literature search was conducted in collaboration with a senior librarian, and CINAHL, Medline, and Embase databases was searched. We had no publication year or date limits for the searches, and the same keywords were used for all the searches. We used Boolean operators (AND, OR), truncation (*) and we searched for words from title (ti.), abstract (ab.), author keyword (kf.) or a single word from the subject headings (hw.). The final searches were conducted on May 16, 2023, with an update on February 15, 2024. In total, 2021 (1848 and 173) matches were identified and eligible for further review.

**Table 1:** Keywords used in the literature search in this study

	Keywords used in the literature search
Leader, leadership	leader*
Team *Ad-hoc teams in hospital, but the search also includes broader aspects of teams (combined with the acute settings)*	team*, acute*, medical*, hospital*, trauma*, ad-hoc*, rapid respons*, code, cardiac, crash, patient care, healthcare, emergen*, critical*, resuscitation*, multidisciplinar*, interdisciplinar* member*, communicat*, teamwork*
Communication	communicat*, miscommunicat*, cues, respond to input, think aloud*, talk*, word*, prompts, speak*, speech*, gaze direction, vocal nuanc*, conversat*, closed loop, facial expression*, gesture*, face expression*, verbal*, nonverbal*, non-verbal*, posture, position*, wordless*
Performance	performance*, effect*, ineffect*, safe, safety, quality of care, outcome*, adverse or harm*, error*, accuracy, completeness, relevance, timeliness, improv*, team cohesion*, evaluat*, succeed, success*
Acute settings	acute*, emergenc*, emergent*, code respons*, rapid respons*, code blue, code team*, blue code, cardiac crash, chrash team*, adhoc, ad-hoc, ad hoc, trauma*, resuscitation*, critical*, cardi*, arrest*, heart arrest*, intensive care, life support*, lifesaving, life saving

*Truncation or shortening of words for an efficient way to search for words with different suffixes

### Eligibility criteria

Studies were included if they (1) involved healthcare professionals in ad hoc teams in a hospital setting, (2) had a quantitative design, and (3) had investigated the association between team leader communication and team outcome. In addition, it had to be published studies written in English.

### Study selection

**Fig. 1:**
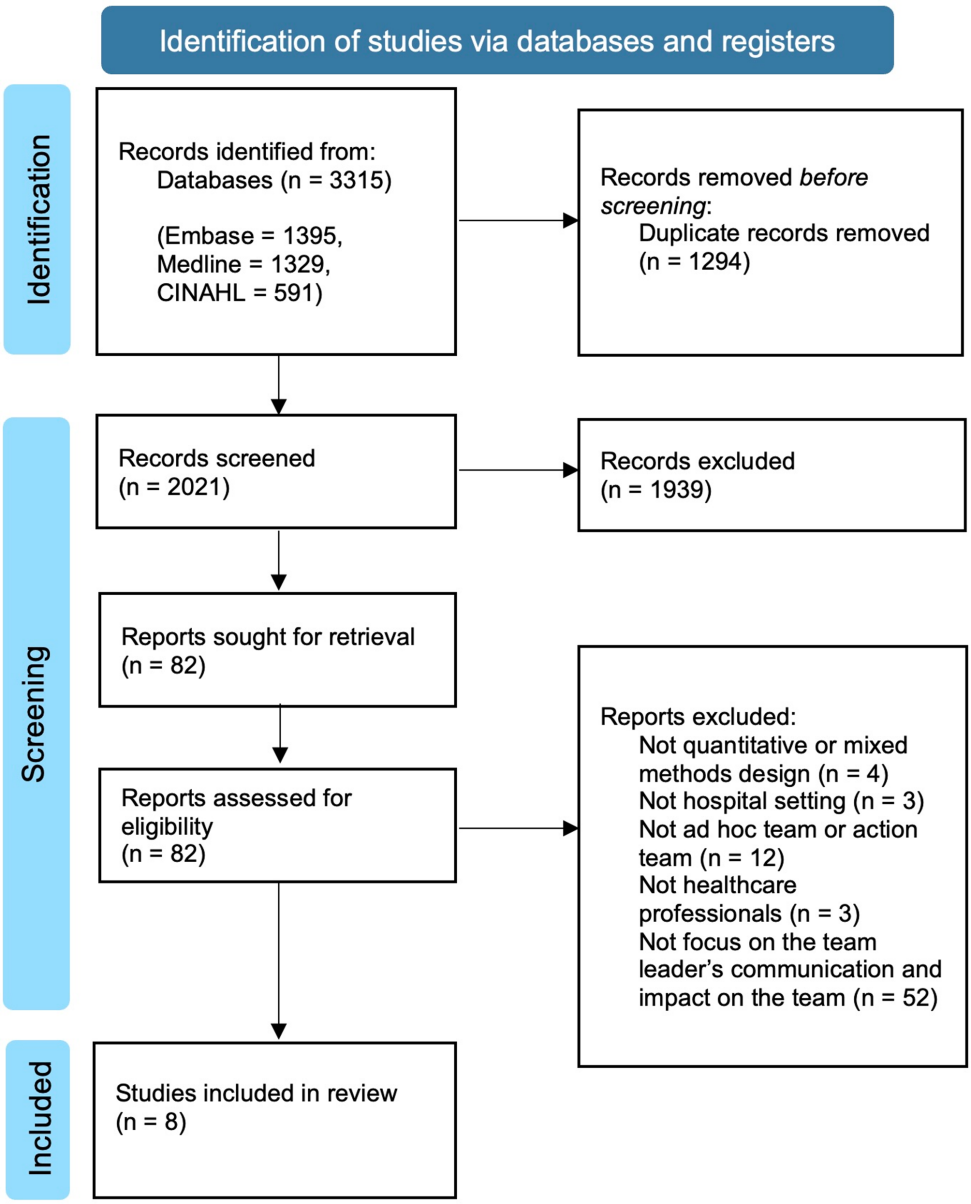
The selection process is illustrated with a PRISMA diagram [40].

The titles, abstracts, and full-text screening of the identified articles were reviewed by three independent reviewers. All the authors discussed any point of disagreement until an agreement was reached. The screening process was conducted using the Rayyan research collaboration platform [39]. Eight articles were included and reviewed. To add depth to the review, citations and reference lists from the eight articles included were also screened. Citations in Google Scholar and Scopus were screened, and reference lists were manually searched. No new articles from the citations or reference lists were included. The search and screening processes are presented in the PRISMA flow diagram shown in Fig. 1 [40].

### Quality assessment

The risk of bias of the included studies was independently assessed by two (ORA and KH) of the authors. As the studies had an observational design, Joanna Briggs Institute’s “Checklist for Analytical Cross-sectional Studies” was used [41]. None of the studies from the review were excluded because of poor quality.

### Data extraction and analysis

All authors read all the included articles to be familiar with the studies. Data extraction tables were constructed to gather relevant information from the studies included. To ensure consistency, one reviewer (ORA) extracted data from the included reports using a pre-designed data recording form, while two other reviewers (DTSJ and KH) verified the extraction for accuracy and completeness. When discrepancies arose, the three reviewers resolved them by re-examining the publication and engaging in further discussion. The data that were extracted were the author names, publication year, aims, settings, study design, measurement methods of team leader communication, and team outcomes. Information on the demographics of the study population, including descriptions of patient cases, team sizes, and professions in the teams, was also collected. Given the diverse measurement methods used in the studies included in this review, a meta-analysis was not possible; therefore, the data were organized into logical categories, presented in tables, and synthesized into a meaningful narrative relevant to the aim of the review [42]. 

## Results

A narrative synthesis of the findings from the eight studies from the literature search was conducted, and the results are presented in the following sections. The characteristics of the studies are presented in Table 2. The team leader communication and the outcomes of the studies are displayed in Table 3.

**Table 2:** Characteristics of the studies included in the review

Source	Context	Patient type	Case type	Teams	Persons in each team	Team composition	Cases	Case descriptions
Briggs 2015 [43]	ER Simulation	Patient simulator	2 trauma scenarios	20	---	Surgery residents, ER residents, ER nurses, and emergency service assistants (nurseʼs aides). One resident was designated as the team leader	35	Scenario 1. Blunt trauma from a motor vehicle accident. Scenario 2. Multiple penetrating injuries from a broken-plate glass window. Both the cases required endotracheal tube and chest tube
Härgestam 2016 [46]	ER In situ simulation	Patient simulator	1 trauma scenario	16	6	One surgeon or ER physician (team leader), one ER nurse, one OR nurse, one RN, one nurse anaesthetist, one anaesthesiologist	--	1 severely injured patient (injury severity score: 25) suffering from hypovolemia due to external trauma
Johnsen 2017 [50]	ER In situ simulation	--	2 trauma scenarios	27	6–7	One surgeon (team leader), one anesthesiologist, one nurse anesthetist, two ER nurses, one lab technician and one radiographer	--	Scenario 1. Adult pedestrian hit by a car Scenario 2. Young construction worker who has fallen 6 m from a scaffold
Abd El-Shafy 2018 [48]	ER Real life	Real patients	Pediatric trauma	--	--	Team leaders were pediatric surgery fellow or ER attending, general surgery resident, an emergency medicine fellow or a resident. No information about the other team members	89	Trauma team activation videos were reviewed; 81 level-II trauma activations and 8 level-I trauma activations
Caldwell 2021 [44]	ER Simulation	MetiMen mannequins	3 trauma scenarioes	13	6 (+2)	18 residents; four PGY2 and five PGY4 in general surgery, nine PGY3 in Emergency Medicine, and two ED nursing staff. Teams selected their own unique resuscitation team leader for each scenario	--	Scenario 1. Young M patient with multi gunshot wound. Scenario 2. Patient involved in MVC due to stroke. Scenario 3. Elderly patient s/p fall with significant LE deformity and brain bleed
Wolfgang 2023 [49]	ER Real life	Real patients	Pediatric non-trauma	--	--	The team leader was a pediatric physician or a fellow or an attendant. Rest of the team: not described	46	Patients with respiratory problems (25%) and shock/sepsis/altered mental status or cardiac arrest (each 18%). 33% received I.V. fluids and 25% received I.V. or I.M. epinephrine. 25% had a critical procedure performed. Median patient age was 6.1 years. 38% were admitted to ICU and eight died in the ER
Lin 2023 [47]	ER and ICU Simulation	Manikin Sim Junior	Pediatric cardiac arrest	20	5	Healthcare providers from ICU and ER. Two trained research actors played the medication nurse and bedside clinician. No information about the team leader	--	1 cardiac arrest scenario
Maiga 2023 [45]	ER Real life	Real patients	Trauma	--	12 (median)	Physician, nurse, resident, fellow or attending The TTL was most frequently assumed by an attendent (43.1%) or resident (40.8%), less commonly a fellow (12%) or advanced practice provider (0.5%).	441	Adult (16 years or older) trauma patients with an initial SBP <90 mm Hg or any episode of SBP <90 mm Hg within the first 5 min of ED presentation.

*ED* Emergency Department, *ER* Emergency Room, *ICU* Intensive Care Unit, *LE* Le Fort injuries that are complex fractures of the midface, *MVC* Motor Vehicle Collision, *PGY2* Postgraduate Year Two, *PGY3* Postgraduate Year Three, *PGY4* Postgraduate Year Four, *RN* Registered Nurse, *s/p* status post

**Table 3:** Results of the studies included in the review

Source	Aim	Study design	Setting	Team leader communication measure	Measurement of team outcome	Team leader`s communication impact on team outcome
Briggs 2015 [43] USA	They hypothesized that team’s and team leader’s NTS correlate with technical performance of clinical tasks.	Retrospective cohort study Observational design Video analysis	Trauma Bay Emergency room Simulation Brigham and Women’s Hospital, STRATUS Center for Surgical Simulation	NTSs performance by team leaders Team leader assessment using the validated NOTSS. 1. Team leadersʼ social skills leadership, (communication, and teamwork) 2. Team leadersʼ cognitive skills (SA and decision making) This validated scoring system is used to assess the NTS performance of a single individual.	NTS of the entire team using the Modified T-NOTECHS examining the relationship between NTSs and technical skill performances. The two 2 teams were also recorded for endotracheal tube (ETT) and chest tube (CT) placement during the scenario: the time that a decision was made to intervene and the time of completion of that intervention.	Team leader`s NTS impact on team performance: Significant Statistics: Cognitive: SA: 0.775 (*p* < 0.001) and decision making: 0.785, p < 0.001), social (communication and teamwork): 0.602 (*p* < 0.001), leadership: 0.657 (p < 0.001) Relation: Strong correlation with team NTS scores, especially in cognitive NTS Cognitive skills’ impact on the critical task completion: Significant Statistics: Cognitive skills (SA *p* = 0.014) (decision making p = 0.039) Relation: Direct correlation with critical task completion Team leader`s NTS impact on secondary task completion: No significance observed. Relation: no significant correlations with secondary task completion
Härgestam 2016 [46] Sweden	To investigate the association between the time taken to a decision to go to surgery and the use of closed loop and leadership styles during trauma team training.	Observational design Video analysis	Emergency room Simulation An urban Scandinavian level 1 trauma center	CLC First by quantifying only CO (only step one included) and then CLC with all three steps included	Primary outcome: The time taken to decision to go to surgery	Leader’s CLC’s impact on likelihood of making a decision to go to surgery within 900 seconds: Significant Statistics: Likelihood of making a decision: HR: 3.88, CI 1.02 to 14.69 Relation: CLC increased likelihood of making a decision
Johnsen 2017 [50] Norway	To investigate the frequency of behavioral markers of SMM demonstrated by team leaders on quality of medical management.	An observational study Video analysis	In the trauma rooms in the teams’ own hospitals. ER Simulation	The ATOM instrument the number of behavioral indicators per minute. Team leaders` frequency of SSM behavioral markers: Information sharing, supporting behavior and SA	A global measure of medical management (quality of medical performance) was performed by two independent SMEs in trauma. Medical management measured only the technical skills exhibited by the trauma teams and was rated on an ordinal 5-point scale from 1 (poor) to 5 (good).	Proactive information sharing’s impact on medical management quality: Significant Statistics: Proactive information sharing: 0.483, *p* < 0.011 Relation: Positive correlation with medical management quality Supportive and updating behaviors’ impact on medical management quality: Significant Statistics: supportive behavior: r (27) = 0.353, *p* < 0.035 (one-tailed); updating: r(27) = 0.437, *p* < 0.030 Relation: Positive correlation with medical management quality Guidance and suggestions’ impact on medical management quality: No significance observed. Relation: No correlation with medical management quality
Abd El-Shafy 2018 [48] USA	To evaluate the ability of CLC to improve time-to-task completion in pediatric trauma activations.	Observational study Video analysis	ER – trauma. Real life An American pediatric trauma center in the New York City area Each trauma room was equipped with 3 ceiling cameras and microphone	The use of CLC and classification all verbal orders issued by the trauma team leader for order audibility and directed responsibility	Reviewers identified and classified all verbal orders issued by the trauma team leader for order audibility, directed responsibility, check-back, and time-to-task-completion. The impact of pre-notification and level of activation on time-to-task- completion was also evaluated.	CLC’s impact on reduction in time-to-task-completion: Significant Statistics: Overall reduction in time-to-task-completion (*p* < 0.0001), orders completed 3.6 times sooner [HR = 3.6 (95% CI: 2.5,5.3)]; Specific reduction in time-to-task: Medication orders (*p* < 0.00221), intravenous line placement orders (*p* < 0.00968), laboratory test orders (*p* < 0.0001), intravenous fluid orders (not significant). Relation: A significant reduction in time-to-task-completion when orders with CLC was used compared to orders without. There was not a significant difference in time-to-task-completion with respect to pre-notification by emergency service providers (*p* < 0.6100). [HR = 1.1 (95% CI: 0.9,1.3)]. There was also not a significant difference in time-to-task-completion with respect to level of trauma team activation (p < 0.2229). [HR = 1.3 (95% CI: 0.8, 2.1)].
Caldwell 2021 [44] USA	Describe the implementation and results of our Multi-Disciplinary Trauma Evaluation and Management Simulation training for GS and EM residents and describe novel tools for evaluation of simulation skills and communication of team leaders.	Observational study	ATLS trauma resuscitation ER Simulation Monitor and score teams in real time from the control room.	Team leaders were evaluated in three domains: 1.evaluation and planning 2.action processes 3.interpersonal skills and team management, - and adapted items from T-NOTECHS, TEAM, and TTCA-24 that directly applied to team leader performance, and which mapped to the ACGME competencies.	Measure of essential medical items completed on the resuscitation checklist items. They developed an instrument which used domains and anchors similar to several prior domains in TEAM, T-NOTECHS, and TTCA-24, focused on specific, actionable feedback (which could be provided to a resident to improve their performance or used to assess resident acquisition of milestones).	Higher communication scores impact on better trauma resuscitation team performance: Significant (in two of three scenarios) Statistics: Scenario 1 and 3: Pearson’s r 0.92 (*p* < 0.01) and 0.95 (*p* < 0.01); Scenario 2 Pearson’s r 0.29 (p = 0.58). Relation: Significant correlation between team leaders who received higher communication scores and the teams’ completion of resuscitation checklist items in scenario 1 and 3, and no significant correlation in scenario 2.
Wolfgang 2023 [49] USA	To describe performance of COs in an emergency setting and to determine whether a CO is associated with better teamwork	Observational study Real life Video analysis	In the pediatric resuscitation area of an academic pediatric ED Not trauma	CO performance by the physician team leader and the use of CLC.	The TEAM tool	CO’s impact on better teamwork (total TEAM score): Significant Statistics: Mean TEAM score: With a CO 42.3 (1.7), without a CO: 40.0 (3.0) (*P* = 0.007). Relation: A significantly better mean team score when CO was performed than those without. No significant effect of CO was observed when controlled for repeated team leader CO’s impact on better teamwork (global team score): No significance observed Statistics: Mean global team score: With a CO 8.74 (1.0), without a CO 8.10 (1.0) (*p* = 0.054). Relation: No significant better global teams score when CO was performed than those without. Team leaders request for team agreements’ impact on teamwork: None significance observed Statistics: request for team agreements (*p* = 0.4898). Relation: Request for team agreement has no significant impact on total team score.
Lin 2023 [47] Canada	To describe the leadership performance of team leaders and CPR coaches, and to determine if a correlation between leadership and CPR performance during management of simulated pediatric cardiac arrest events.	Observational study An 18-minute simulated cardiac arrest scenario Video analysis	The ER and ICU of 4 tertiary care pediatric hospitals Simulation Secondary analysis of data from a previous large multicenter RCT	The BAT to assess leadership performance of the team leader and the CPR coach. Coordinating initiation of CPR, providing corrective verbal feedback and reinforcement of chest compressions, coordinating defibrillation, switches, and intubation.	CPR quality parameters including CC depth (mm), CC rate (cc/min) and chest compression fraction for the whole simulation scenarios were collected. Compliance with 2020 AHA guidelines were defined as: depth 50–60 mm and rate 100–120/min. The percentage of overall excellent CPR, defined as meeting AHA guidelines for CC depth and rate at the same timeand (ii) Chest compression fraction.	Team leaders’ impact on CPR team performance: Significant on excellence; none on CCF Statistics: Association with excellent CPR (Spearmans’s r = 0.52, *p* = 0.02) and CCF (r = 0.22, *p* = 0.35) Relation: A significant association between team leader BAT and the percentage of excellent CPR performance, and no association between team leader BAT and CCF. CPR coaches’ impact on CPR team performance: Significant on CCF; positive association (not significant) on excellent CPR Statistics: Association with CCF (Spearman’s r = 0.48, *p* = 0.03) and excellent CPR (r = 0.390, *p* = 0.09) Relation: A significant association between CPR coach BAT and the percentage of CCF, and between CPR coach BAT and CCF
Maiga 2023 [45] USA	We hypothesized better team performance scores to be associated with decreased time to the next phase of trauma care.	A multicenter prospective observational study Video analysis	Nineteen trauma centers Multicenter study Real life	Leadership in a modified T-NOTECHS: communication and teamwork, assessment and decision making and SA	Time to next phase of trauma care Inpatient mortality	Better leadership and communication`s impact on faster timed to next phase of care: Significant Statistics: Independent adjusted T-NOTECHS score for better leadership (*p* < 0.05) and better team leader? communication (p < 0.05) Relation: A better leadership score (e.g. team leader clearly recognizable, delegation, assigns roles, transitions of leadership clear) and better communication score (e.g. team leader is the hub, critical communication through the team leader, CLC, orders directed to specific people) were associated with faster time to next phase of care. Neither the overall T-NOTECHS team performance score nor any of the individual T-NOTECHS subset scores were found to be associated with inpatient mortality. Only ISS and initial HR

*ACGME* Accreditation Council for Graduate Medical Education, *AHA* American Heart Association, *ATOM* The Anti-Air Teamwork Observation Measure, *BAT* The Behavioral Assessment Tool, *CC* chest compression, *CCF* chest compression fraction, *CI* Confidence Interval, *CLC* closed-loop communication, *CO* Call-outs, *CPR* cardio pulmonal resuscitation, *ED* Emergency Department, *EM* emergency medicine, *ER* Emergency Room, *GS* general surgery, *HR* Hazard Ratio, *HR* Heart Rate, *ISS* Injury Severety Score, *NOTTS* non-technical skills of surgeons, *NTS* non-technical skills, *p* significance level, *r* correlation ratio, *RCT* randomized controlled Trial, *SA* situational awareness, *SMM* shared mental model, *TEAM* Teamwork Emergency Assessment Measure, *T-NOTECHS* Nontechnical Skills for Trauma, *TTCA-24* Trauma Team Communication Assessment

### Team training in advance of data collection

Five of the articles described some form of team training, although none reported on specific team leader training. In a study by Briggs and colleagues [43] trauma team training faculty provided an introduction to crisis resource management before the initial scenario. Caldwell and colleagues [44] outlined a brief, 30-minute didactic session focused on advanced trauma life support (ATLS) trauma resuscitation delivered to residents before simulations. Meanwhile, Maiga and colleagues [45] documented two comprehensive ATLS training sessions prior to the study’s start. Härgestam and colleagues [46] explored teams in which many members had previously received team training. Lin and colleagues [47] referred to a previous randomised controlled trial (RCT) intervention that included 1-hour training sessions for CPR coaches while other participants watched a 5-minute orientation video on using CPR feedback. Participants in the intervention arm viewed an additional 1-minute video about the CPR coaching concept. Lastly, a study by Abd El-Shafy and colleagues [48], which focused heavily on closed loop communication (CLC), did not detail prior training but cited the ATLS and Team STEPPS programmes as relevant frameworks.

### Measurement of team leader communication

The measurement of team leader communication varied across the studies. Four studies used validated measurement instruments to measure team leader communication [43–45, 47]. Four studies measured behavioural indicators to assess team leader communication [46, 48–50], of which three measured team leaders’ use of CLC [46, 48, 49] and one used the anti-air teamwork observation measure to count the frequency of specific behavioural indicators per minute [50]. 

### Impact of team leader communication on team outcomes

All the results of the studies demonstrated the significant impact of team leader communication on team outcomes, as shown in Table 3. The outcomes were measured as technical work performance and non-technical work performance, both of which were considered teamwork performance.

### Team leader communication’s impact on teams’ technical work performance

Four of the studies included in the review investigated the impact of team leader communication on technical work performance. A significant correlation between situational awareness (SA) and team leader decision-making and critical task completion was identified [43]. Where SMM and the use of SA were investigated, a positive correlation was found between the quality of medical treatment and team leader communication. Specifically, leaders who shared information spontaneously without explicit requests and who communicated their SA through implicit supportive behaviours demonstrated enhanced treatment quality [50]. 

A non-trauma simulation study conducted in the EDs and ICUs of four paediatric hospitals found that higher team leader scores were significantly associated with a higher percentage of excellent CPR, while no association was found between team leader scores and chest compression fraction [47].

Four of the studies measured team leaders’ impact on the time required to complete critical clinical tasks. A large multicentre study conducted in a real life context found that the team leaders’ and the entire team’s NTS (leadership, SA, team communication, assessment and decision-making) scores were associated with faster times to the next phase of care [45]. The three other studies that measured time as a team outcome measured the impact of CLC use by the team leader. A study that investigated paediatric trauma activations in a real life context found a significant overall reduction in time to task completion when CLC was used. Orders with CLC were completed 3.6 times sooner than orders with open loop communication. The examined orders were medication, intravenous line placement, laboratory tests and intravenous fluid orders, with the latter not demonstrating a significant result [48]. In a study conducted in a real life context in a paediatric trauma bay, time to task completion was significantly reduced when CLC was utilised [48], and CLC initiated by the leader increased the likelihood of making the decision to go to surgery in another study [46]. Briggs and colleagues [43] measured time to task completion in their trauma simulation study and found a significant association between team leaders’ NTS scores and clinical task completion. A trauma study from a paediatric ER in real life that investigated all verbal orders issued by the trauma team leader and time to task completion found a significant reduction in time to task completion when closed loop communication was utilised. Orders with CLC were completed 3.6 times sooner than orders with an open loop [48]. 

### Team leader communication’s impact on teams’ non-technical performance

The relationship between team leader communication and non-technical teamwork performance was investigated in two studies [43, 49]. One study on paediatric resuscitation in real life investigated the impact of structured callouts (COs) [49]. Team leaders performed COs in 63% of patient encounters, and 40% of COs included a request for agreement from the team. Only 6% received a response. CLC is recommended as a standard for safe communication in acute care, and a CO without a request for response and confirmation is not a complete CLC [51]. However, the performance of COs in this study was significantly associated with better teamwork, as measured by the teamwork emergency assessment measure (TEAM) score. A trauma simulation study found a significant correlation between the team leader’s communication and the entire team’s non-technical skills scale for trauma (T-NOTECHS) scores, and the strongest correlation was noted in the team leaders’ situational awareness and decision-making scores [43]. 

## Discussion

The aim of this review was to investigate whether team leader communication in an ad hoc team impacts the performance of the entire team. The outcome in the studies was categorized in two types of measures, technical work performance and non-technical work performance. All studies showed a significant association between team leader communication and team performance.

This review found that team leaders’ communication showed a strong correlation with technical work performance, in one study technical work was based on essential medical items from a scenario checklist [44]. Adherence to procedures and protocols is a highly regarded measurement method in this research area [2]. Cardiac arrest resuscitation has shown that team leader communication has an impact on CPR performance [30, 47]. Cooper and colleagues [30] found that when a team leader initiated a structure, the team members were more likely to perform resuscitation correctly and at the right time, while leaders with hands-on participation were less likely to lead effectively. The studies in this review did not report hands-on time. However, it would be interesting to investigate this in relation to team leadership in ad hoc teams. Tschan and colleagues [52] investigated the correlation between the performance scores of electrical cardioversion and performance during CPR in ED simulation, finding a significant positive correlation between CPR and leaders’ statements that made the team aware of the current situation. Leaders’ clear communication and statements about SA using CO may impact the clinical task performance of the entire team and contribute to positive team outcomes.

In addition to CPR teams, team leader communication also impacts trauma teams’ technical performance, as demonstrated in the studies included in this review. The team leader’s use of CLC, which showed a positive impact on the time from arrival to the decision to take the patient to surgery [45] and a reduction in time to task completion [48], may be significant to patient outcomes. This can have a lifesaving effect, as time is crucial when the patient’s condition is critical. Trauma resuscitation time is associated with effective damage control surgery and remains the cornerstone of trauma management [53]; therefore, time to surgery is a good team outcome measure and facilitates ensuring patient safety. The use of COs is a demanding leadership style that may be necessary during acute crises in ad hoc teams. Furthermore, CLC has been the most frequently reported team leadership behaviour in facilitating team members’ input and speaking-up behaviors [16]. Speaking-up behaviours flourish in psychologically safe teams, and the use of speak-up and CLC may contribute to clarifying intended messages, thereby preventing errors. A study showed that 51% of errors in paediatric trauma resuscitation were never acknowledged or compensated for by the team [48]. Team leaders must be trained to use this simple and effective communication tool, tools that are commonly used in other fields, such as aviation and the military [54]. 

Whether it is non-technical or technical work, there are certain inputs that can have an impact on team processes [8]. To have team leader competence, the team leader must be trained, as dedication and willingness are not enough. Systematic team training as an input in a hospital or unit may impact the process and, in turn, the outcomes [55]. In most studies in this review, the team leader was a resident. Residents are physicians with little experience who may not have been trained in leadership in their medical studies. Teams directed by surgeons with low leadership competency took significantly longer to complete the key steps in the initial trauma patient evaluation [56]. Team leadership should be a part of medical and nursing curricula and education and team training in teamwork is recommended for continuing education training, in which the healthcare organisation’s management is responsible for. To invest in team training has been done with good results in other studies [32, 55, 57–59], however, more research on team leader training is needed [32]. In six of the included articles in this review, some kind of team training or didactics was described; however, it was limited to very brief sessions.

Previous reviews advocated for the development and implementation of team training [32, 36] with an impact on patient outcomes [55]; however specific team leadership training needs more focus [32]. As for leadership in aircrews, special training programmes were developed early on and have become a standard for which many other high-risk industries are still striving [60]. There is a need for the further development of training concepts embedded in leading in healthcare that focus especially on leadership in ad hoc teams.

Having a team leader trained in the use of leadership and communication strategies can make a difference to the outcomes, both for team performance and for patient safety and patient outcomes [30]. Only one study in this review measured patient outcomes directly by measuring inpatient mortality [45]. However, the team leader communication in the ad hoc teams did not impact mortality, which may have been due to the study size and the design of the study.

In a real life study on paediatric resuscitation in the ED, the researchers found that CO, which is designed to capture the immediate attention of the entire team, was associated with better teamwork performance. The team leader’s use of communication strategies, such as CO and CLC, mediates the process of teamwork, which impacts team performance in a positive way [8]. However, only a few COs in this study resulted in the completion of CLC [49]. In contrast, the teams with the least COs had the highest number of CLCs, which can mean that COs should be used only when necessary, such as when critical and valuable information must be shared with the entire team.

Briggs and colleagues [43], who measured the time from ED arrival to when a decision was made to intervene, found no significant associations between team leader’s general NTS scores and the time taken for any of the technical work tasks. This may indicate that it is more appropriate to measure team leaders’ use of a specific communication tool, such as CLC, to find correlations with team outcomes. However, both verbal and non-verbal behaviours, such as frequent question-asking and behavioural mimicry, are also recommended for leader evaluation and have been shown to have positive effects on team performance [61].

With the growing understanding of the importance of NTS alongside clinical and technical skills, it is important to investigate elements that mediate the impact of leadership on team performance and patient safety [35]. Effective communication in teams, especially when initiated by team leaders, can prevent errors and adverse events arising from misunderstandings and a lack of SA [2]. The critical importance of non-technical skills, such as leadership, communication and teamwork arise from aviation safety and accident investigation, and which has a link to patient safety [62]. In two of the studies in this review, non-technical work performance was the team performance outcome measure [43, 49]. One was conducted in a real life context [49] and the other was a simulation study [43], and both studies showed positive outcomes. Regarding the measurement of NTS in ad hoc teams, there has been an inconsistency in the definitions and measurement of the NTS constructs; to reconcile these inconsistencies, Evans and colleagues [63] developed a taxonomy of NTS, which they recommended for use in future ad hoc team research.

Situation awareness (SA), that was positively associated with technical work performance in Johnsen and colleges [50] has also been applied in other fields such as aviation [64]. A previous review found a significant impact of SA on technical skills for team performance as well as some non-technical skills, such as leadership, task management, mindfulness and task prioritisation and concluded that few studies have investigated the use of SA within the context of hospital emergency care [65]. Hoven and colleagues [66], who investigated team leadership in aviation teams, found that leaders who were less effective in establishing SA had the least effective teams.

Another mediator shown to impact team performance is psychological safety in teams [67], however this aspect of team leadership was not studied in any of the included studies in the current review. Psychological safety, which is nurtured by leaders through an inclusive leadership style, empowers team members to believe they can speak up about concerns or ideas without fear of punishment, rejection or embarrassment. This is essential for ensuring patient safety and effective teamwork outcomes [68]. Leadership with a willingness to listen to others and foster a climate that increases the chance of members speaking up about issues is strongly recommended and is a mediator in the teamwork process, with a positive impact on team performance and patient safety [67]. The opposite is passive communication with a fear of speaking up [69]. Riuco and colleagues [69], who studied leadership and communication styles of airline pilots, found that effective team leader communication, described as a participative leadership style with assertive communication, had a positive impact on crew member’s satisfaction, however, they did not study impact on team performance.

Many variables can be used as outcome data measures, such as compliance with protocols, errors and mortality rates are useful; however, they all have both benefits and drawbacks [70]. Several studies in this review used more than one measurement method, which is recommended because it can reduce the biases associated with relying on one source of measurement [71]. Compared with inputs and outcomes, the mediator, as team leader communication, may be difficult to observe in real time studies, since team processes must be observed while the teams are performing a task. Therefore, the most common data collection methods used are video reviews from real life or simulation, as in these studies [70]. Video observation may provide valid data on the team process and is more accurate than self-reported data from questionnaires collected retrospectively [71]. 

This review contributes to the large body of teamwork research in healthcare by pointing out the importance of the team leader’s communication and use of communication tools and its impact on the entire team’s performance. In this way, we extend previous reviews focusing on team leader style by specifying the impact on team performance of the communication of the team leader. We have demonstrated that the team leader’s communication in ad hoc teams has a positive impact on the team’s performance, and team leaders are advised to have competence in team leader communication and to be aware of the importance of their responsibilities. A team leader with calm and clear communication who uses callouts and closed loops and is able to establish SA may contribute to preventing misunderstandings and communication failures, thereby preventing errors and adverse events that can have fatal consequences for the patient [2]. 

### Strengths and limitations

This study has both strengths and limitations. One strength is that the search strategy was broad and was performed in cooperation with an experienced librarian. Another strength is that eligibility was independently screened by three reviewers at each screening phase, and a consensus among the review team was reached if there was doubt about the inclusion of an article.

One limitation might be that some relevant studies were missed because this study focused only on quantitative studies published in English. Initially, we wanted to conduct a meta-analysis, but during the inclusion process, this was not possible because there were no studies with an RCT design; therefore, we ended up with only observational studies. Although all the articles met the inclusion criteria of team leader communication in ad hoc teams and were quite similar in team size, composition and context, the measurement methods varied across the studies. These factors limit the ability to draw strong conclusions based on the existing evidence in this review. More rigorous studies with stronger designs are needed to investigate how team leader communication in ad hoc teams can impact team outcomes.

## Conclusions

The results of this review indicate that the team leaders of ad hoc teams have a significant impact on team performance and that team leader communication has a positive impact on the non-technical and technical work performance of the entire team. We found that team leaders’ use of communication strategies, such as situational awareness, callouts and closed loop communication, positively impacted team performance. To be an effective leader that manages patient situations that are often unpredictable and in which every minute counts requires a competent team leader.

This review underscores the importance of incorporating team leader training into team training programmes and educational curricula, emphasising team leader communication and its impact on team performance. Further studies using more comparable measurement methods for both team leader communication and team performance are needed to confirm the results of this review. The findings in this review may contribute to an enhanced emphasis on the training of team leaders in intense, unpredictable situations, such as in ad hoc teams.

## Data Availability

The literature search history may be available from the corresponding author upon reasonable request.

## Abbreviations

ATLS: Advanced trauma life support
CLC: Closed loop communication
CPR: Cardiopulmonary resuscitation
ED: Emergency department
ICU: Intensive care unit
NTS: Non-technical skill
OR: Operating room
PRISMA: Preferred Reporting Items for Systematic Reviews and Meta-Analyses
SA: Situational awareness
SMM: Shared mental model
TEAM: Trauma Emergency Assessment Measure
T-NOTECHS: Non-Technical Skills for Trauma
